# Geographic Expansion of *Baylisascaris procyonis* Roundworms, Florida, USA

**DOI:** 10.3201/eid1611.100549

**Published:** 2010-11

**Authors:** Emily L. Blizzard, Michael J. Yabsley, Margaret F. Beck, Stefan Harsch

**Affiliations:** Author affiliations: University of Georgia, Athens, Georgia, USA (E.L. Blizzard, M.J. Yabsley);; Goose Creek Wildlife Sanctuary, Tallahassee, Florida, USA (M.F. Beck);; SPCA Wildlife Care Center, Ft. Lauderdale, Florida, USA (S. Harsch)

**Keywords:** parasites, worms, Baylisascaris procyonis, ascarids, nematodes, raccoons, Florida, zoonoses, expedited, letter

**To the Editor:** *Baylisascaris procyonis* roundworms are common parasites of raccoons (*Procyon lotor*) in several regions of North America, Europe, and Asia. These parasites are increasingly recognized as a cause of larva migrans in humans, an infection that often results in severe neurologic sequelae or death. In addition, larva migrans has been documented in ≈90 species of wild and domestic birds and mammals. In the United States, *B. procyonis* roundworms are most prevalent in the midwestern, northeastern, and Pacific western states. Numerous surveillance studies have been conducted in the southeastern United States, and *B. procyonis* roundworms are most common in the mountainous regions of Virginia, Kentucky, and West Virginia (*1*–*4*). Geographic expansion of *B. procyonis* roundworms has been recently documented in Georgia. In 2002, 22% of raccoons sampled in DeKalb County, Georgia, a highly urbanized area near Atlanta, were positive for the parasite (*5*), and recently, 10% of raccoons sampled in Clarke County, Georgia, were positive (*6*). Whether this expansion is due to natural spread of the parasite among raccoons or to translocations of infected raccoons into naive areas is unclear. We document expansion of *B. procyonis* roundworms into northwestern and southeastern Florida.

In 2006 and 2007, nine ascarids (>3 inches) were collected from the feces of an unrecorded number of raccoons admitted to a rehabilitation center in northern Florida. In September 2008, December 2009, and June 2010, one ascarid each was found in the feces of a 4- and a 6-month-old raccoon from Leon County, Florida, and a 6-month-old raccoon from Wakulla County, Florida, after routine treatment with pyrantel pamoate (20 mg/kg). In July 2010, a juvenile (6-month-old) raccoon from Broward County, Florida, which had been admitted to a rehabilitation center, passed several ascarids (2 collected for testing) in its feces after ivermectin treatment (0.2 mg/mL) for mange. The 14 ascarids were preserved in 70% ethanol, and adult males were identified as *Baylisascaris* spp. on the basis of their morphologic characteristics (perianal rough patches). The ascarids were subsequently confirmed as *B. procyonis* by sequence analysis of the 5.8S rRNA gene or the internal transcribed spacer (ITS)-1 and ITS-2 regions (*7*,*8*). The complete sequences of the 5.8S rRNA gene and ITS-2 region from 2 ascarids from northern Florida and 1 from southern Florida were identical to *B. procyonis* sequences (GenBank accession nos. AJ001501 and AB051231, respectively). ITS-1 sequences from the 2 ascarids from northern and southern Florida were 99.1% (424/428; AB053230) to 100% identical (AJ00745 and ascarids from Georgia, Kentucky, and Texas [*6*]), respectively, to *B. procyonis* sequences.

Several previous studies did not detect *B. procyonis* roundworms in raccoons or latrine sites in central Florida (n = 51 from Glades, Highlands, Hillsborough, and Orange counties), southern Florida (n = 90 from around Miami and n = 64 fecal samples on Key Largo), and numerous counties throughout Florida (n = 177) (*1*,*3*,*9*). Historically, *B. procyonis* roundworms have been absent throughout most of the Southeast, but the parasite was recently detected in north-central Georgia (*5*,*6*). How the species became established in Florida remains unclear. Establishment could have resulted from natural dispersal of infected raccoons from *B. procyonis*–endemic areas; however, recent examination of several raccoon populations in southern Georgia failed to detect such infections (*6*). Alternatively, the parasites could have been introduced from the movement of infected raccoons, exotic pets (e.g., kinkajou [*Potos flavus*]), or natural wildlife intermediate hosts (*1*).

Additionally, because domestic dogs can serve as definitive hosts, an infected dog from a *B. procyonis*–endemic area may have passed eggs into the environment (*1*). Veterinarians in Florida should be aware of this possible zoonosis and carefully examine ascarid eggs detected in fecal specimens because *B. procyonis*–infected dogs often have mixed infections with *Toxocara canis,* *Toxascaris leonina*, or both, which have morphologically similar eggs (*1*). Physicians, veterinarians, and wildlife biologists in Florida should be aware of this serious pathogen and the likelihood its range will increase, as highlighted by the recent detection of *B. procyonis* roundworms in a kinkajou from southern Florida (K.R. Kazacos et al., unpub. data).

This study also highlights the importance of wildlife rehabilitation centers as resources for the study of wildlife/zoonotic diseases. Animals admitted to rehabilitation centers are often ill or injured, which may increase pathogen shedding or transmission. Additionally, young raccoons are likely to be infected with *B. procyonis* roundworms, and kits as young as 3 months of age can be patent. Numerous fatal *B. procyonis* larva migrans infections have occurred among animals in rehabilitation centers and zoological parks. These infections were likely acquired when animals were housed in enclosures previously occupied by infected raccoons or when bedding or food became contaminated with *B. procyonis*–infected raccoon feces. In *B. procyonis*–endemic areas, cages used to house raccoons should be thoroughly decontaminated by flaming, or cages should be dedicated for use by raccoons. Because *B. procyonis* roundworms can spread to other animals, persons in contact with raccoons should be alert to potential transmission routes and apply appropriate biosecurity procedures.
